# The Role of FKBPs in Complex Disorders: Neuropsychiatric Diseases, Cancer, and Type 2 Diabetes Mellitus

**DOI:** 10.3390/cells13100801

**Published:** 2024-05-08

**Authors:** Galila Agam, Bayan Atawna, Odeya Damri, Abed N. Azab

**Affiliations:** 1 Department of Clinical Biochemistry and Pharmacology, Faculty of Health Sciences, The Zlotowski Center for Neuroscience and Zelman Center—The School of Brain Sciences and Cognition, Ben-Gurion University of the Negev, Beer-Sheva 8410501, Israel; atawnab@post.bgu.ac.il (B.A.); odeyad@post.bgu.ac.il (O.D.); 2 Department of Nursing, School for Community Health Professions, Faculty of Health Sciences, Ben-Gurion University of the Negev, Beer-Sheva 8410501, Israel

**Keywords:** cancer, complex disorders, *FKBP5*, neuropsychiatric disorders, type 2 diabetes mellitus (T2DM)

## Abstract

Stress is a common denominator of complex disorders and the FK-506 binding protein (FKBP)51 plays a central role in stress. Hence, it is not surprising that multiple studies imply the involvement of the FKBP51 protein and/or its coding gene, *FKBP5*, in complex disorders. This review summarizes such reports concentrating on three disorder clusters—neuropsychiatric, cancer, and type 2 diabetes mellitus (T2DM). We also attempt to point to potential mechanisms suggested to mediate the effect of *FKBP5*/FKBP51 on these disorders. Neuropsychiatric diseases considered in this paper include (i) Huntington’s disease for which increased autophagic cellular clearance mechanisms related to decreased FKBP51 protein levels or activity is discussed, Alzheimer’s disease for which increased FKBP51 activity has been shown to induce Tau phosphorylation and aggregation, and Parkinson’s disease in the context of which FKBP12 is mentioned; and (ii) mental disorders, for which significant association with the single nucleotide polymorphism (SNP) rs1360780 of *FKBP5* intron 7 along with decreased DNA methylation were revealed. Since cancer is a large group of diseases that can start in almost any organ or tissue of the body, FKBP51’s role depends on the tissue type and differences among pathways expressed in those tumors. The FKBP51–heat-shock protein-(Hsp)90–p23 super-chaperone complex might function as an oncogene or as a tumor suppressor by downregulating the serine/threonine protein kinase (AKt) pathway. In T2DM, two potential pathways for the involvement of FKBP51 are highlighted as affecting the pathogenesis of the disease—the peroxisome proliferator-activated receptor-γ (PPARγ) and AKt.

## 1. Introduction

In this review we intend to examine the hypothesis that FK-506 binding proteins (FKBPs) are hubs in complex disorders. To this end, we will summarize what the FKBPs are, what complex disorders are, and to which extent the information existing in the literature supports the notion that FKBPs are hubs in complex disorders.

## 2. The *FKBP5/FKBP4* Genes and Their Products FKBP51/52, Respectively 

The FK506-binding protein 51 (FKBP51) is encoded by the *FKBP5* gene. These proteins (FKBPs) act as cis–trans peptidyl-prolyl isomerases (PPIase), belonging to the immunophilins protein family [1,2]. Most of the FKBPs are effective in binding immunosuppressive drugs [2]. FKBP51/52 were initially discovered in association with steroid hormone receptors, glucocorticoid receptors (GRs), or progesterone receptors (PRs). The assembly undergoes through a specific pathway involving heat-shock proteins (Hsp)40, Hsp70, Hsp90, and various Hsp-binding cochaperones [3].

The roles of the larger FKBPs are less understood, but FKBP51 and FKBP52 have emerged as likely contributors to health and disease, in particular, for their potential as therapeutic targets for the treatment of a variety of hormone-dependent diseases including stress-related disorders, immune and reproductive functions, and a variety of cancers [4,5,6,7]. FKBP51 and FKBP52 are involved in physiological processes both in the brain and in the periphery, including binding to immunosuppressants like tacrolimus (FK506) and rapamycin, protein folding and trafficking, inhibition of calcineurin, and regulation of steroid hormone receptor maturation and translocation to the nucleus [4,5,6,7].

Under normal and stress conditions, FKBP51 functions as a cochaperone of HSP90 [8,9,10] and modulates inflammation through nuclear factor κB (NF-κB) signaling [11,12]. Genetic variants and epigenetic modifications of glucocorticoid-responsive genomic sites regulate the *FKBP5* gene. The interplay of these factors, along with interactions with environmental stressors, may lead to FKBP51 disinhibition, potentially contributing to abnormal phenotypes observed in both rodents and humans [13].

In most studies, FKBP51 is observed as a negative regulator of steroid hormone receptor activity, while FKBP52 positively regulates the activity of most of the steroid hormone receptors, except for estrogen receptor/mineralocorticoid receptor activity [14,15,16,17]. 

These alterations in biological functions are likely associated with structural domains, e.g., critical residues for PPIase activity are conserved in FKBP52 and FKBP51 [4,5], but residues within the proline-rich loop, positioned above the PPIase pocket, differ between them, significantly impacting protein interactions with larger peptide substrates. Co-immunoprecipitation studies have shown that the motor protein complex dynein and subunit components of dynactin bind to the N-terminus of FKBP52, seemingly independent of FKBP52’s PPIase activity [4,5,18]. Conversely, FKBP51 delays the nuclear translocation of GR, possibly due to its weak interaction with dynein [6,7,16,19].

## 3. Complex Disorders, Stress, and *FKBP5* and Its Products

Complex disorders are characterized by a combination of genetic, environmental, and lifestyle factors, many of which remain unidentified. This category of diseases encompasses a range of conditions, including congenital defects, adult-onset diseases, neuropsychiatric disorders, type 2 diabetes mellitus, and cancer [20]. Stress has been demonstrated to play a role in all these complex disorders [21,22,23], and *FKBP5*’s gene variants have been demonstrated to play a role in stress-related phenotypes and disorders [24].

### 3.1. Neuropsychiatric Diseases

Neuropsychiatric diseases include a broad range of medical conditions in which the central nervous system (CNS) is involved—both neurological diseases and mental disorders. Neurological diseases include seizures, attention deficit disorders, cognitive deficit disorders, palsies, migraine headaches, Alzheimer’s disease, Huntington disease, amyotrophic lateral sclerosis, Parkinson’s disease, and more. 

Mental disorders (MDs) refer to conditions characterized by behavioral impairments that result in distorted personal functioning [25]. The affective (emotion/mood) disorders and anxiety categories represent the core classifications within the spectrum of MDs. The former involves profound and enduring experiences of sadness, melancholy, or despair, as observed in unipolar depression, as well as bipolar disorder (BD), marked by alternating phases of abnormally elevated, normal, and depressive mood states. The latter encompasses conditions such as phobias, generalized anxiety, social anxiety, and post-traumatic stress disorder (PTSD). MDs also include disorders mainly presenting psychotic features (e.g., delusions and hallucinations) such as schizophrenia. A prevalence exceeding one in three individuals worldwide indicates the experience of at least one episode of a MD at some stage in their lives [26]. 

### 3.2. Type 2 Diabetes Mellitus (T2DM) 

T2DM is characterized by high blood glucose, insulin resistance, and a relative lack of insulin [27]. It is a chronic disease with a ten-year shorter life expectancy due to complications including significantly higher risk of cardiovascular disease/ischemic heart disease, stroke, and lower limb amputations as well as increased rates of hospitalizations [28].

### 3.3. Cancer 

Cancer comprises a diverse group of diseases marked by the rapid development of abnormal cells that surpass their normal confines, infiltrating neighboring tissues and disseminating to other organs. To comprehend the intricacies of cancer biology, it is imperative to explore the underlying capabilities facilitating tumor growth and metastatic spread. These capabilities include sustaining proliferative signaling, evading growth suppressors, enabling replicative immortality, activating invasion and metastasis, inducing angiogenesis, and opposing cell death [29]. 

Multiple studies imply a central role of FKBPs in multiple complex disorders. This review summarizes such reports and points to potential mechanisms mediating these roles [7,8,10,30,31,32,33].

## 4. Methods

The strategy used to prepare this review was to search for relevant manuscripts written in English using electronic databases such as *PubMed*, published between 1947 and present, with either of the keyword combinations “FKBP5 and mental disorders”/“FKBP5 and cancer”/“FKBP5 and type 2 diabetes mellitus”. The first two were limited to article types: Clinical Trials, Meta-Analysis, and Randomized Controlled Trials. In PubMed, about 35 research articles were found to be suitable. Furthermore, the reference lists of these articles were used to enrich the review. Data extracted from the included papers were initially collated by the second author (BA) in a Microsoft Word document, summarizing general characteristics and the intervention design in a structured manner. The third author (OD) verified the data. The corresponding authors resolved discrepancies and concluded the review.

## 5. Involvement of *FKBP5*/FKBP51 in Neuropsychiatric Diseases

### 5.1. Neurological Disorders

#### 5.1.1. Huntington’s Disease (HD)

Bailus et al. established the implication of FKBP51 in the pathogenesis of HD, highlighting *FKBP5* as a potential therapeutic target for intervention [34]. Current HD-modifying therapies focus on lowering mutant HTT (huntingtin; mHTT) levels. In this context, rapamycin interacts with *FKBP5*/FKBP51 (and *FKBP1A*/FKBP12, see below under Parkinson’s disease), inhibiting the mTORC1 complex and increasing cellular clearance mechanisms. The study showed significantly reduced FKBP51 protein levels in HD R6/2 and zQ175 mouse models, in human HD isogenic neural stem cells, and in medium spiny neurons derived from induced pluripotent stem cells. Furthermore, FKBP51 interacted and colocalized with HTT in the striatum and cortex of zQ175 mice and controls. Importantly, in the isogenic human HD stem cell model, genetic or pharmacological manipulations which reduced FKBP51 protein levels and activity-resulted in decreased mHTT levels. These interventions revealed that increased levels of the autophagy-associated protein LC3-II and macroautophagic/autophagic flux followed the decrease in FKBP51 protein levels, suggesting that autophagic cellular clearance mechanisms are responsible for mHTT lowering [34].

#### 5.1.2. Alzheimer’s Disease (AD)

Multiple studies investigated the involvement of FKBP51 (as well as FKBP52) in AD [35,36,37,38,39,40]. For example, Jinwal et al. showed that FKBP51 interacts with Tau, a protein predominantly found in neurons, which, in healthy brains, forms part of and stabilizes microtubules [41]. In brains of AD’s patients, Tau is misfolded. Downregulation of FKBP51 activity (using siRNA) profoundly decreased Tau expression. Blair et al. [42] reported that over-expression of the chaperone complex consisting of FKBP51 and HSP90 in mice inhibited proteasomal degradation of Tau, leading to Tau accumulation. Additionally, they showed that FKBP51 levels age-relatedly increase in human brains, particularly in subjects with AD. Importantly, they found a positive correlation between FKBP51 levels and the severity of AD. Similarly, Giustiniani et al. [43] demonstrated a strong interaction between FKBP52 and a specific Tau mutant (Tau-P301L) that is frequently linked to the pathophysiology of AD. FKBP52 induced Tau-P301L aggregation and, contrastingly, diminution of FKBP52 activity inhibited Tau-P301L accumulation and restored axonal growth and branching in a transgenic zebrafish model. In a follow-up study, Giustiniani et al. [44] observed that FKBP52 interacts with Tau through a specific proline-rich region. FKBP52 inhibited microtubule growth and led to the oligomerization and aggregation of Tau. Consistent with these findings, FKBP52 over-expression was shown to result in increased Tau phosphorylation and aggregation in aged mice. The over-expression of FKBP52 was also associated with the activation of glia cells and neuronal loss and led to a decrease in hippocampal volume. The aforementioned biochemical changes were accompanied by different behavioral abnormalities in the aged mice [45]. Taken together, the summarized data suggest that FKBP51 and FKBP52 induce Tau phosphorylation and aggregation leading to typical AD-related pathological morphological changes in the brain.

#### 5.1.3. Parkinson’s Disease (PD)

**Figure 1:**
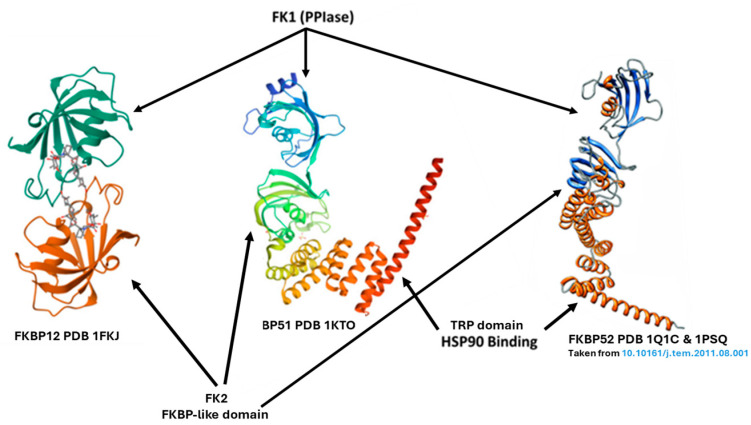
The 3-D structure of FKBP12 vs. FKBP51 and FKBP52. Left: Atomic structure of FKBP12-FK506 (https://doi.org/10.2210/pdb1FKJ/pdb, accessed on 29 April 2024). FKBP12 contains the PPIase core domain but not the C-terminal tetratricopeptide repeat (TPR) domain that mediates interaction with Hsp90. Middle: structure of the Large FKBP-like Protein, FKBP51 (https://doi.org/10.2210/pdb1KT1/pdb, accessed on 29 April 2024). Right: Structure of FKBP52 [7]. FKBP51 and FKBP52 share 70% similarity and contain an active PPIase domain, bind Hsp90 through a TPR domain [3], and adopt similar formations.

Interestingly, our literature search for an involvement of *FKBP5*/FKBP51 in PD failed to obtain such information. However, it was found that another partner of the FKBP family is associated with PD (and AD). While FKBP51 is the largest homolog in the family of FKBPs, the members of which possess a highly conserved binding pocket but have diverged to perform diverse biological functions, FKBP12, with a molecular weight of 12 kDa, is the smallest member of the FKBP family [46] (Figure 1).

FKBP12 also contains the PPIase core domain, but it does not contain the TPR domain that mediates interactions with Hsp90 [47]. It occurs in most species and tissues and is essential for mammalian life. It is a cofactor of the ryanodine receptor [48], thereby playing a role in fine-tuning the excitability of smooth muscle cells and cardiac myocytes [49]. Pertinent to the current review, it is noteworthy that the enzymatic activity of FKBP12 promotes the formation of α-SYN fibrils at sub-nanomolar concentrations [50,51]. α-synuclein (α-SYN), a presynaptic protein prone to aggregation, is associated with α-synucleinopathies, including PD. Honjo et al. [52] reported that FKBP12 colocalized with α-SYN in Lewy bodies (LBs, the hallmark of PD and dementia with Lewy bodies (DLB) and neurites in PD and DLB brains. FKBP12’s strong aggregation function is compatible with the notion of the protein’s association with synucleinopathies’ pathophysiology.

### 5.2. Mental Disorders

#### Genetics and Epigenetics of *FKBP5*

The FKBP51 protein emerges as a risk factor for various affective disorders. Coordinating with Hsp90, it plays a pivotal role in regulating GR activity through a concise negative feedback loop. This signaling pathway swiftly reinstates homeostasis within the hypothalamic–pituitary–adrenal (HPA) axis in response to stress. The upregulation of *FKBP5* expression over time is attributed to diminished DNA methylation associated with aging. Elevated levels of FKBP51 correlate with GR resistance and a diminished capacity for stress coping behaviors [10]. Additionally, common allelic variants within the *FKBP5* gene are linked to increased susceptibility to MDs such as depression, anxiety, and PTSD [10].

Research on *FKBP5*/FKBP51 has experienced significant growth since the revelation that polymorphisms in this gene have the potential to influence treatment outcomes and depressive behavior in humans. The number of studies conducted on *FKBP5*/FKBP51 has more than doubled since this pivotal discovery [53]. This coincided with additional data indicating that the stress hormone axis plays a role in the onset of various mental illnesses. Genome-wide association studies (GWAS) focusing on single nucleotide polymorphisms (SNPs) have uncovered noteworthy associations between allelic variants of the *FKBP5* gene and mental disorders [53].

In a study that delved into the interplay of polymorphisms and epigenetic targets within intragenic regions [54], it was proposed that epigenetic alterations and environmental factors can shape the impact of allele-specific variants, influencing the response of the *FKBP5* gene to glucocorticoids. The primary focus of epigenetic investigation in *FKBP5* intronic regions has been on DNA methylation, although only a limited number of studies have explored this across all GRs in these regions. A significant discovery has been the association of decreased DNA methylation levels in intron 7 of *FKBP5* among individuals with psychiatric disorders [54]. Furthermore, *FKBP5’s* DNA methylation has been implicated in the response to exposure-based psychological therapy for phobias [55]. Flasbeck and Brüne [56] showed that GR sensitivity is regulated by *FKBP5*’s methylation and correlates with psychopathology and empathy scores but not with the severity of childhood adversity.

A Chinese study [57] reported a negative association between a history of childhood physical and emotional neglect and DNA methylation in the promoter region of the *FKBP5* gene across most CpG units. Specifically, the study identified child abuse as a risk factor for mood disorders and demonstrated a connection to reduced DNA methylation of *FKBP5* intron 7, particularly through interactions with the SNP rs1360780 [58]. Another study suggested that the *FKBP5* gene’s polymorphisms contribute to the risk of depressive disorders and suicidal behavior [59]. Larger studies are needed to validate these results.

Saito et al. [60] investigated the impact of the interaction between a specific subtype of child abuse and the *FKBP5* rs1360780 SNP on DNA methylation in individuals with bipolar disorder. Their findings indicated that emotional abuse and/or neglect were associated with reduced DNA methylation of *FKBP5* intron 7, particularly in conjunction with the rs1360780 SNP. No such results were obtained in major depression disorder patients or in control subjects.

Roberts et al. [55] reported changes in DNA methylation at *FKBP5* intron 7 to be associated with reduced anxiety severity following exposure-based cognitive behavioral therapy. A noteworthy correlation with follow-up outcomes was identified concerning the alteration in DNA methylation during active therapy (from pre- to post-treatment) at CpG 5 of the intron. Those exhibiting reduced DNA methylation at this CpG site demonstrated a more favorable response to treatment, as evidenced by a higher improvement in the Clinical Global Impression severity scale. Conversely, individuals with increased DNA methylation exhibited a less favorable treatment outcome. To investigate allele-specific effects of DNA methylation, the impacts of interactions between the rs1360780 SNP in *FKBP5* and changes in each CpG site on treatment outcomes were assessed. Intriguingly, a nominally significant interaction between the rs1360780 genotype and changes in DNA methylation at CpG 2 of the promoter region was observed. Subsequent exploration of this effect revealed that individuals with the “risk” genotype (CT/TT) displayed a poor treatment response with a decrease in percentage DNA methylation, whereas an increase was associated with a more favorable response.

The combination of bullying and a certain *FKBP5* haplotype was associated with increased positive psychotic-like experiences, paranoia, and negative affect. This interaction also intensified the link between feeling socially rejected and psychotic-like experiences and negative affect in daily life, particularly in individuals with the risk haplotype [61] but not for those with the non-risk haplotype. Epigenetic changes are now being considered as potential explanations for the persistence and resilience of PTSD and major depression disorder, a complex condition characterized by diverse and often unique responses to treatment [62,63,64,65].

Using data from target bisulfite sequencing of the whole *FKBP5* locus, a very recent study [66] analyzed associations between *FKBP5* methylation, *FKBP5* mRNA levels, childhood maltreatment, and current depressive symptoms. The nominally significant results obtained might supplement the understanding of the role of *FKBP5*. The authors conclude that, given its regulating role in the HPA stress response, *FKBP5* could serve as a biomarker or as a target for developing therapeutic interventions for stress-related disorders such as depression. Furthermore, based on previous studies reporting that alterations in methylation patterns are reversible via psychotherapy [67,68] and that methylation markers may predict the outcome of therapy [69,70], the authors suggest that in-depth understanding of epigenetic mechanisms involved in depression may facilitate the development of personalized treatment and prevention.

An additional recent study that investigated the antidepressant activity and mechanism of rosmarinic acid [71], a natural phenolic acid compound with a variety of bioactive properties, reported that the compound’s antidepressant activities evolve by modulating hippocampal glucocorticoid signaling and hippocampal neurogenesis, related to the BDNF/TrkB/PI3K signaling axis regulating GR nuclear translocation.

### 5.3. Involvement of FKBP5/FKBP51 in Cancer

*FKBP5* exhibits altered expression levels in a variety of tumors. By impacting the maturation of steroid receptors and influencing NF-κB and AKt signaling pathways, *FKBP5* assumes a crucial role in tumorigenesis and the response to anti-neoplastic therapy [72]. The dual role of *FKBP5*, whether functioning as an oncogene or a tumor suppressor, may be contingent on the tissue type and variations among the pathways expressed in those specific tumors. For example, *FKBP5* was found to be downregulated in pancreatic tumor tissue, while it is over-expressed in melanoma [72]. Activation of *FKBP5* transcription via the androgen receptor (AR) was reported in prostate cancer [73,74] and via the GR in human lung cancer A549 cells [75].

#### 5.3.1. The FKBP51–Hsp90–p23 Super-Chaperone Complex

In addition to the Hsp90-FKBP51 interaction mentioned above, a coordinated interaction between the ATP-utilizing chaperone HSP-90 and the cochaperones FKBP51 and p23 produces an FKBP51–Hsp90–p23 super-chaperone complex that promotes androgen-dependent transcription activation and cell growth. Thus, FKBP51 is part of a positive feedback loop regulated by AR and androgen. Hence, downregulation of its levels results in reduced transcript levels of genes regulated by AR and androgen [76].

#### 5.3.2. FKBP51 Regulation of the NF-κB Pathway

NF-κB is a transcription factor existing in all cell types, mostly existing in the cytoplasm in an inactive form as it is bound to the inhibitor I-κB [77]. Upon phosphorylation via either the canonical or noncanonical pathways [78], NF-κB is released from IκB and is translocated to the nucleus where it upregulates the transcription of specific genes [27]. FKBP51 is essential for drug-induced NF-κB activation in human leukemia [79].

#### 5.3.3. FKBP51 as a Negative Regulator of the AKt Pathway

As mentioned above, Akt plays an important role in cell death, growth, and division; apoptosis suppression; and angiogenesis. Hence, disruptions in the AKt-regulated pathways are associated with cancer [80]. FKBP51 is a negative regulator of all three isoforms of AKt, AKt1, AKt2, and AKt3, which are considered central mediators of the insulin signaling pathway [81,82,83].

Protein phosphatases have been shown to dephosphorylate AKt while FKBP51 functions as a scaffolding protein that enhances the PH domain and leucine-rich repeat protein phosphatase (PHLPP)–AKt interaction and facilitates PHLPP-mediated dephosphorylation of AKt-Ser473 [72]. Protein phosphatase 2 (PP2) dephosphorylates Ser308 [84], while PHLPP1 and 2 dephosphorylate Ser473 [85]. FKBP51’s involvement in cancer aggressiveness and cell growth has been reported in multiple human malignancies. As eloquently and comprehensively summarized by Tufano et al. [86], dysregulated FKBP51 sustains tumor resistance and growth. On the other hand, FKBP51 might also function as a tumor suppressor in the AKt signaling pathway. Namely, high FKBP51 levels lead to decreased AKt phosphorylation and, therefore, increased chemosensitivity [86].

### 5.4. Involvement of FKBP5/FKBP51 in T2DM

T2DM patients were reported to exhibit elevated *FKBP5* expression in subcutaneous adipose tissue with an inverse relationship between the gene’s expression and the expression of lipolytic, lipogenic, and adipogenic genes [87]. On the other hand, another study on peripheral blood mononuclear cells derived from patients with T2DM showed reduced levels of *FKBP5* mRNA, phosphorylated GR protein content, and elevated GRβ. These findings suggest inadequate GR signaling and dysfunction of the HPA axis [88]. Furthermore, in mice exposed to a chronic high-fat diet, which induces insulin resistance, hypothalamic *FKBP5* expression was found to be enhanced, indicating that *FKBP5* senses metabolic stressors including nutrient environment [89]. Described above is the role of *FKBP5* DNA methylation in MDs. It is also relevant in T2DM. *FKBP5* DNA methylation correlated with adiposity, insulin resistance, and systemic inflammation; it inversely correlated with the gene’s mRNA levels and was positively associated with adiposity, metabolic, and inflammatory parameters [90]. In addition, increased *FKBP5* DNA methylation correlated with higher hemoglobin (Hb)A1c levels [91].

Considering the above-mentioned negative regulation of AKt2 via FKBP51, it is not surprising that the ability of insulin to lower blood glucose was impaired in *Akt2*-deficient mice, due to defects in the action of insulin on liver and skeletal muscle [82].

Ortiz et al. [91] hypothesized that *FKBP5* methylation is associated with potential glucocorticoid pathway dysfunction as an underlying pathophysiology of diabetes, hyperglycemia, hyperlipidemia, obesity, and cardiometabolic disease. Their hypothesis was based on the following points: *FKBP5* and the GR are expressed in the brain (hypothalamus and hippocampus), intestinal endothelial, and epithelial tissues. *FKBP5* expression in these tissues has been associated with the influence of stress and cortisol load, exercise, and diet; *FKBP5* also plays a role in adipogenesis and in endothelial changes. In individuals with diabetes, *FKBP5* methylation is associated with an increased incidence of clinical risk factors for disease.

Alternatively, FKBP51 inhibition via compounds that bind it, such as its specific inhibitors SAFit1 or FK506, may directly downregulate adipogenesis and lipid storage, increase insulin sensitivity, and reduce body mass [87,92].

An additional molecular mechanism that has been suggested for the role of FKBP51 in T2DM is the positive regulation of PPARγ, the main regulator of adipocyte differentiation and function. When wild-type (WT) and *FKBP5*-knockout (KO) mice maintained on regular and high-fat diet conditions were compared, the KO mice were resistant to weight gain, hepatic steatosis, and had significantly reduced amounts of white- but higher brown-adipose tissue. When rosiglitazone (a PPARγ agonist used as an anti-diabetic drug) was administered, it induced whole-body weight gain and an increased mass and elevated expression of PPARγ-regulated lipogenic genes in the white adipose tissue of WT mice. On the other hand, the drug reduced these parameters in the KO mice [93]. For further information, the readers are encouraged to refer to the recent review by Smedlund et al. [94].

## 6. Conclusions

**Figure 2:**
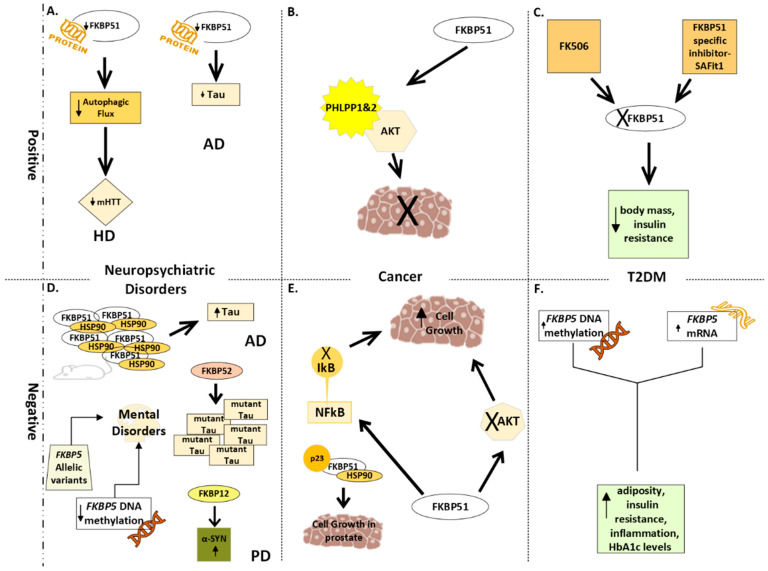
An illustration of the involvement of FKBPs in complex disorders. Generally, increased FKBP51 enzymatic activity/protein levels and/or *FKBP5* mRNA levels are related to negative effects, namely, increased disease severity (and vice versa). Positive effects: (**A**) Neuropsychiatric diseases—in HD, when FKBP51 protein levels or activity were decreased using genetic or pharmacological approaches, reduced mHTT levels were observed in the isogenic human HD stem cell model [24]. In AD, downregulation of FKBP51 activity (using siRNA) profoundly decreases Tau expression [49]. (**B**) Cancer—FKBP51 may function as a tumor suppressor in the AKt signaling pathway, through a scaffolding protein that enhances the PH domain and leucine-rich repeat protein phosphatase (PHLPP)–AKt interaction [76,78]. (**C**) TD2M—compounds that bind/inhibit FKBP51, such as FK506 and the specific inhibitor SAFit, may directly diminish adipogenesis and lipid storage, increase insulin sensitivity, and reduce body mass [80,85]. Negative effects: (**D**) Neuropsychiatric diseases—in relation to AD, over-expression of the chaperone complex consisting of FKBP51 and HSP90 in mice inhibited proteasomal degradation of Tau and led to Tau accumulation [50]. FKBP52 interacts with Tau through a specific proline-rich region. FKBP52 inhibited microtubule growth and led to oligomerization and aggregation of Tau [52]. In relation to PD, enzymatic activity of FKBP12 increases the formation of α-SYN fibrils at sub-nanomolar concentrations [59]. In relation to psychiatric disorders, low DNA methylation levels were reported in *FKBP5*’s intron 7 in patients with psychiatric disorders [61] and common allelic variants in this gene were reported to be associated with increased risk of developing affective disorders (anxiety, depression, and PTSD) [18]. (**E**) Cancer—*FKBP5* functions as an oncogene or a tumor suppressor depending on the tissue type and the pathways expressed in those tumors. For example, activation of *FKBP5* transcription via the androgen receptor was reported in prostate cancer [67,68]; an FKBP51–Hsp90–p23 super-chaperone complex stimulates androgen-dependent transcription activation and cell growth [70]; FKBP51 is essential for drug-induced NF-κB activation in human leukemia [73]; and FKBP51 was identified as a negative regulator of Akt [75,76]. (**F**) TD2M—*FKBP5* DNA methylation correlated with adiposity, insulin resistance, and systemic inflammation; it also inversely correlated with *FKBP5* mRNA levels, which were positively associated with adiposity, metabolic, and inflammatory parameters [82]; increased *FKBP5* DNA methylation correlated with higher HbA1c levels [83]. Abbreviations: AD—Alzheimer’s disease; AKt/PKB—RAC-alpha serine/threonine-protein kinase/protein kinase B; HD—Huntington’s disease; HSP90—heat-shock protein 90; IκB—inhibitor of κB; mHTT—mutant huntingtin; NF-κB—nuclear factor kappa-light-chain-enhancer of activated B cells; PD—Parkinson’s disease; PHLPP—Pleckstrin homology (PH) domain leucine-rich repeat protein phosphatase; α-SYN—alpha-synuclein; T2DM—Type 2 diabetes mellitus. 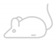 A mouse model of PD. 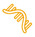 mRNA. 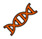 DNA. 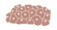 Fast proliferating cells (cancer). 
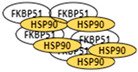
 Over-expression of the chaperone complex consisting of FKBP51 and HSP90. 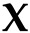 Inhibition. 
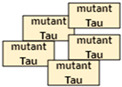
 Aggregation of mutant Tau.

Figure 2 depicts a summary of the reviewed studies illustrating that *FKBP5*/FKBP51 and *FKBP1A*/FKBP12 play a role in complex disorders [95]. In general, the FKBP51 protein is considered a risk factor of MDs and a potential therapeutic target, given the significant relationship between its high levels and MDs development [96]. In particular, high levels of FKBP51 are linked to an increased risk of mood disorders [10]. Studies on *FKBP5* knockout mice and inhibition of FKBP51 suggest that inhibiting FKBP51 could alleviate depressive symptoms without affecting cognition or movement [97,98]. *FKBP5* methylation in mice correlates positively with chronic stress in the blood and brain [99,100]. Human studies indicate elevated FKBP51 levels in the lymphocytes of depressed individuals with common *FKBP5* gene variations, associated with a quicker response to antidepressants but more frequent depressive episodes [101]. Conversely, FKBP51 expression is reduced in whole blood from PTSD patients [102]. Obviously, understanding the link between peripheral FKBP51 levels and brain function in neuropsychiatric disorders requires further study. Developing selective, FKBP51-targeting drugs is challenging due to similarities with other FKBP proteins like FKBP52. A recent discovery, SAFit2, a selective FKBP51 antagonist, holds promise in enhancing neuronal function and stress coping [98,103]. Yet, more research is needed to fully understand SAFit2’s effects and explore alternative therapies targeting FKBP51. FKBP51 is an important regulator of stress in mammals and, therefore, it is possible that some of the compounds inspired by the early immunophilin ligands will be useful for the treatment of stress-related disorders. Concerning T2DM, Balsevich and colleagues [32] demonstrated that FKBP51 antagonism enhanced glucose tolerance, with weight loss being a secondary factor. In other words, the primary cause of improved glucose tolerance is FKBP51 antagonism rather than weight loss itself, which can also contribute to improved glucose tolerance. Regarding cancer, studies have indicated that using FK506, which binds to the FK1 domain of FKBPs, or MJC13, an inhibitor of FKBP52, to treat prostate cancer can inhibit cell proliferation and AR activity [104,105,106]. As for human lung cancer, it has been suggested that FKBP51 could serve as a biomarker. Additionally, the authors report that FKBP51 over-expression induces apoptosis in vitro by promoting p53 expression and signaling pathway activation [107]. In both cancer and T2DM, the role of FKBP51 in the pathogenesis of the diseases depends on the specific pathways involved [75].

Building on the data summarized, we assert that both *FKBP5* and FKBP51 may be designated a hub in complex disorders, but definitive physiological mechanisms mediating their effects require further studies [104,105,106]. Genetic/environmental/lifestyle factors affect complex disorders, making treatment challenging. In this respect, comprehending the impact of FKBP5 on disorder susceptibility necessitates the integration of genetic, epigenetic, and transcriptional analyses, offering potential assistance in diagnosis and treatment. A relevant option might be manipulating the *FKBP5* and/or *FKBP1A* genes or their respective protein products FKBP51 and FKBP12. Newly developed selective FKBP51 blockers have shown encouraging results in vitro and in rodent models [13]. As such, the recently discovered transient rearrangement of the binding pocket of FKBP51 [107] might provide specificity.

## Abbreviations

AD	Alzheimer’s disease
AKt	Serine/threonine protein kinase
AR	Androgen receptor
FKBP	FK-506 binding protein
GR	Glucocorticoid receptor
HD	Huntington’s disease
HPA	Hypothalamic–pituitary–adrenal
Hsp	Heat-shock protein
HTT	Huntingtin
KO	Knockout
mHTT	Mutant huntingtin
PD	Parkinson’s disease
PHLPP	PH domain and leucine-rich repeat protein phosphatase
PPARγ	Peroxisome proliferator-activated receptor-γ
PTSD	Post-traumatic stress disorder
SNP	Single nucleotide polymorphism
α-SYN	α-synuclein
Tau	Tubulin associated unit
T2DM	Type 2 diabetes mellitus
α-SYN	α-synuclein
Tau	Tubulin associated unit
T2DM	Type 2 diabetes mellitus
